# (In)visible and (Un)heard? Older Adults as Guests on COVID-Related Political Talk Shows in Germany

**DOI:** 10.1093/geroni/igac009

**Published:** 2022-03-02

**Authors:** Janina Myrczik, Catherine Bowen, Annette Franke, Leonie Täuber, Eva-Marie Kessler

**Affiliations:** 1 Department of Psychology, MSB Medical School Berlin, Berlin, Germany; 2 Department of Social Work, Protestant University of Applied Sciences Ludwigsburg, Ludwigsburg, Germany

**Keywords:** Age discrimination, COVID-19, Frame analysis, Mass media, Social participation

## Abstract

**Background and Objectives:**

The coronavirus disease 2019 (COVID-19) pandemic has disproportionately affected older adults. Despite calls to include older people in societal decision making, the extent to which older adults have participated in the pandemic-related public debate is unknown. This study investigated older adults’ (65+ years) voice and visibility as guests on political talk shows as an important arena of public debate. Specifically, we examined how often older adults appeared as guests, their characteristics, and how older versus younger guests discussed the pandemic.

**Research Design and Methods:**

Judges assessed all guests’ age, gender, migration experience, functional aids, and professional background on all episodes of the 4 most-watched political talk shows in Germany between January 1 to December 31, 2020 (*N* = 136 episodes, *K* = 754 guests). We used an exploratory sequential mixed-methods approach and frame analysis on all episodes featuring older guests (*n* = 37), to first identify how guests discussed the pandemic, and then assess differences in positions between older and younger guests (<65 years).

**Results:**

Older guests rarely appeared (12.2% of all guests, 9.6% of guests on COVID-related episodes) and if they did, they were majorly male, young-old, German-born professionals with no functional aids. Rather than appearing as “peer advocates” of older adults, older guests framed the pandemic similarly to younger guests, with a tendency to more strongly address disproportionate restrictions of civil liberties in society.

**Discussion and Implications:**

Results suggest that one prominent part of German media failed to represent the diverse voice of a population most affected by the COVID-19 pandemic in 2020. Differences between how older and younger guests discussed the pandemic may reflect their privileged background in addition to generational differences in attitudes toward government. Future research in other social fora and of other social groups of older adults might facilitate understanding how older adults shaped the public debate on the COVID-19 pandemic.

**Translational Significance:** The coronavirus disease 2019 pandemic has posed a particular threat to older people’s rights and social participation. Using the sample case of German political talk shows, our results suggest that older adults have not extensively participated in the public debate surrounding the pandemic despite being a strongly affected demographic. By demonstrating the under- and homogenous representation of older people, our results have the potential to encourage media producers to better represent the diversity of older people’s needs, perspectives, and competencies. Our findings may thus reduce intersectional ageism in the media and ultimately promote older people’s agency and social participation.

## Background

Over the past few decades, there has been increasing political recognition of older people’s human rights, their diverse needs, and competencies, as well as the societal obligation to include older persons in societal decision making at all levels (United Nations, 2002; WHO, 2020). In the coronavirus disease 2019 (COVID-19) pandemic, older adults aged 65+ years have been affected disproportionately as they are more likely to get very sick, be hospitalized, and die from the SARS-CoV-2 (Severe acute respiratory syndrome coronavirus type 2) virus (e.g., Center for Disease Control and Prevention, 2021). Further, older individuals in many countries faced many of the very strict measures designed to contain the disease, such as bans on visits in long-term care facilities (Martins Van Jaarsveld, 2020). Moreover, ageist narratives in the COVID-19 pandemic experienced a sharp increase with negative effects on older adults’ self-perception (Kornadt et al., 2021) and life satisfaction (Schlomann et al., 2021). Given the extent to which they have been affected by the pandemic, it is highly important for older people to actively participate in the public discourse on the COVID-19 pandemic. However, scholars widely agree that the COVID-19 pandemic might have posed a threat to older people’s agency, autonomy, and active contributions to society, and caused a strong marginalization of older adults (for an overview, see Silva et al., 2021). These views suggest that older adults’ citizenship rights installed to avoid marginalization and systematic disengagement have been undermined by ageist policies and public narratives.

Against this background, this study examines older adults’ voice and visibility as guests on COVID-related political talk shows in Germany in 2020, a year deeply marked by the COVID-19 pandemic. Germany has one of the oldest populations in the world (20.9% aged 65+ years; Federal Statistical Office of Germany, 2020), and COVID-related mortalities have been high primarily due to the proportion of the population aged 80+ years (Federal Statistical Office of Germany, 2021b). Political talk shows are an important and influential arena for public debate and citizen engagement, particularly in Germany (Kessler & Lachenmeier, 2017). As political talk shows are also a mass media outlet, older people’s frequency of appearance on political talk shows also serves as an indicator of older people’s participation and power in society (Harwood & Roy, 2005). Our examination of political talk shows in Germany is thus ideal for examining the extent to which older people have participated in the pandemic-related public discourse, as well as how older people themselves have used their voice in the debate.

### Older People’s Social and Media Participation Before and During the COVID-19 Pandemic

Social gerontology stresses the importance of appreciating the diverse needs, resources, and competencies of older persons, and includes older persons as active cocreators of society (United Nations, 2002; WHO, 2020). However, a plethora of studies has documented that older people are excluded from mainstream society in many ways (e.g., with regard to civic participation, social relations, financial resources; Walsh et al., 2017). Further, the intersectionality of age with other characteristics (e.g., gender, functional health) can put certain subgroups of older people at even greater risk of marginalization. For example, older women, older ethnic minorities and migrants, older people with a health impairment as well as older people living in nursing homes, and the “oldest old” (generally defined as people aged 80+ or 85+ years old; Ihle et al., 2016) are at even greater risk of being excluded (Walsh et al., 2021).

The marginalization of older people is reflected—and also reinforced—by the representation of older people in the media (Loos & Ivan, 2018). Older people—and older women and the oldest old in particular—are remarkably absent from magazines, television series, and advertising, and predominantly portrayed as vulnerable, lonely, and dependent (Kessler et al., 2004; Ylänne, 2015). Parallel to the increasing political recognition of older people’s rights and resources, older people have become somewhat more visible in the media over the past two or three decades, and increasingly portrayed as exceptionally active, vital, and competent when appearing as individual persons (Kessler et al., 2010; Ylänne, 2015).

It is widely assumed that the COVID-19 pandemic has (re)stimulated the societal “othering” of older people in society. Many scholars from the field of gerontology have argued that policies to mitigate the disease have neglected the heterogeneity of older people, and/or ignored older adults altogether (e.g., Ayalon et al., 2021; Kessler & Bowen, 2020). Studies on newspapers and social media have provided preliminary evidence that the homogenous, one-sided negative stereotype of the vulnerable old person has (re)emerged during the pandemic (Allen et al., 2021; Allen & Ayalon, 2021; Bravo-Segar & Villar, 2020; Jimenez-Sotomayor et al., 2020; Köttl et al., 2021; Lichtenstein, 2021; Martikainen & Sakki, 2021; Xiang et al. 2021), with only some studies finding a more differentiated portrayal and counternarratives to these stereotypes (Barrett et al., 2021; Köttl et al., 2021; Xi, 2021). With regard to their participation in the pandemic-related debates, older adults are thought to have “been silent or silenced” during the COVID-19 pandemic (Falvo et al., 2021). In the absence of empirical data, however, the extent to which older adults have participated in the public debate surrounding the pandemic remains speculative.

It is often assumed that older adults’ participation in decision-making processes will ensure that the needs and perspectives of older adults are taken into account (United Nations, 2002). Due to the absence of empirical data, it is, however, still unclear which positions older people adopt in the public discourse toward the COVID-19 pandemic, and whether they express more concerns about the situation of the older population than their younger peers.

So far, few studies have compared how older and younger adults think about the pandemic. Existing studies on people’s personal attitudes toward the COVID-19 pandemic have been limited to how older and younger adults react to measures designed to contain the virus, and their results have been mixed. While one study found that older people experienced more ambivalence than younger people toward measures (Falvo et al., 2021), in other studies, older adults were less likely to comply with measures (Clark et al., 2020), or showed even more compliance (Machida et al., 2020). Moreover, studies found that older adults sometimes oppose policies that would in fact benefit older people and hence themselves due to internalized age stereotypes (Levy & Schlesinger, 2005). There is no evidence that older people advocate for their age peers to a greater extent than younger people. Above all, there are no studies on how older adults voice public positions toward the COVID-19 pandemic.

## The Present Study

So far, little is known about how older people have participated in the public debate surrounding the COVID-19 pandemic. Our study therefore examined the participation of older adults in pandemic-related debate on the four most-watched political talk shows in Germany in 2020 (January–December). Political talk shows have a highly relevant impact on public opinion formation in Germany (Kessler & Lachenmeier, 2017). Around 15% of all German television viewers watch political talk shows each week (Quotenmeter, 2021), and newspapers often pick up the debates (Roth, 2015). The market share of political talk shows in Germany increased further during the COVID-19 pandemic (Mantel, 2020), indicating their decisive role for the pandemic-related debate. As part of public broadcast in Germany, talk shows are obliged to provide citizens with balanced information (Roth, 2015).

### Research Aims and Hypotheses

We had three study aims. First, we were interested in how often older guests—and particularly the oldest old, older women, older migrants, and older people with functional impairment—appeared as guests on political talk shows. We therefore examined the proportion of older guests (65+ years) on COVID-related episodes relative to the proportion of older guests on episodes unrelated to the pandemic and relative to the proportion of older adults in the German population. We also examined older guests’ age (young-old vs old-old), gender, migration experience, and whether they had a functional aid. In concert with findings on the social participation and media representation of older adults, we expected that older adults—and particularly the oldest old, older women, older migrants, and older people with functional impairments—would be underrepresented on political talk shows relative to the population. However, due to the increased focus on older adults as a risk group during the pandemic, we expected the underrepresentation of older people to be less extreme in COVID-related episodes. Our hypothesis is consistent with Froehlich and Hillje (2020), who found talk shows during the COVID-19 pandemic featured more science and fewer political experts than before the pandemic, indicating that producers have adapted the selection of guests due to the pandemic.

As a second study aim, we examined older guests’ professional background. Generally, talk show guests are invited according to their thematic expertise as well as their entertainment value and media compatibility (Meyer et al., 2000). Guests are primarily politicians, but also often journalists, economists, celebrities, as well as everyday persons (Kessler & Lachenmeier, 2017). We expected that most older guests would be professional experts as opposed to laypeople in line with previous research on media depictions of older people (Kessler et al., 2010; Ylänne, 2015) as well as previous research on political talk show guests in Germany (Froehlich & Hillje, 2020). We had no hypotheses regarding older guests’ specific domains of expertise.

Our third study aim was to compare *how* younger and older guests discussed the COVID-19 pandemic. We used an exploratory sequential mixed-methods design (Creswell & Plano Clark, 2018) to first identify distinct ways the pandemic was discussed on the talk shows, and then compare how frequently different approaches were used by older versus younger guests. We also compared how often older versus younger guests explicitly discussed the situation of older people during the pandemic. Due to the absence of studies on public positions of older adults in addition to mixed findings of studies on how older versus younger adults think personally about protective measures enacted during the pandemic, we had no fixed hypotheses about whether older and younger guests would approach the pandemic differently in public statements. Similarly, in light of research on subjective perceptions of age and aging, we did not expect that older guests would address the situation of older adults during the pandemic any more than younger adults.

## Method

### Sample

We analyzed all episodes of the four political talk shows in German television with the highest audience numbers (Mantel, 2020) broadcast between January 1, 2020 and December 31, 2020 (*N*_total_ = 136 episodes). The shows *Maybrit Illner*, *Anne Will, hart aber fair* with a share in the market of around 15% and *maischberger.die Woche* with a share of 10% (Mantel, 2020).

### Data Collection and Coding

#### COVID-related episodes

The first, the fourth, and the senior author used the descriptions on the shows’ official websites to determine whether the episode was related to COVID-19. Episodes were classified as COVID-related if the majority of the descriptions mentioned COVID.

#### Chronological age

The first and fourth authors consulted the guest’s Wikipedia entry or—if unavailable—conducted a web-based search for the date of birth. If only the year of birth was given, we assumed the guest’s birthday had already taken place in 2020. We found age information for 97.9% of the guests. There was substantial agreement between coders, Cohen’s κ = 0.93 (95% confidence interval [CI]: 0.80–1.1), *p* < .001. We recruited 10 independent judges (age range: 22–60 years old) with no knowledge of the study aims to estimate the age of the 16 guests where the web search did not deliver the exact age. Judges used individual screenshots from the relevant episode to estimate each guest’s exact age. Agreement across judges was very high, intraclass correlation coefficient = 0.97 (95% CI: 0.9–0.97). We classified guests older than 65+ years as “older” (OECD, 2021). As in other studies (Ihle et al., 2016), we classified older guests as either *young-old* (65–79 years) or *old-old* (80+ years).

#### Gender

The first and the fourth authors classified guests’ gender based on the German-language gender-marker suffix used in the guest description on the show’s website (e.g., in German *Politiker* indicates a male politician, *Politikerin* indicates a female politician). There was perfect agreement between the two judges, Cohen’s κ = 1 (95% CI: 1–1), *p* < .001.

#### Migration experience

Two independent judges used the results of a web-based search to determine whether guests had migration experience (e.g., living in Germany, born in a different country). There was moderate agreement between the two judges, Cohen’s κ = 0.59 (95% CI: 0.50–0.69), *p* < .001.

#### Functional aid

Two independent judges watched the episodes and recorded whether guests had a hearing aid, walking stick, armband, wheelchair, or walker. There was moderate agreement between the two judges, Cohen’s κ = 0.72 (95% CI: 0.43–1), *p* < .001.

#### Professional background

The senior and fourth authors used the episode descriptions on the shows’ official websites to classify guests’ professional background. We adapted existing role categories of political talk show guests (Fröhlich & Hillje, 2020) to the topic of COVID-19. The role categories were: *politics, science, journalism, state/bureaucracy, civil society (e.g., nongovernmental organizations for older people), culture, economy, social/education, medicine, care,* summarized as experts, and *layperson.* Whenever the guest’s description mentioned multiple role categories (*k* = 11 guests), the first and the senior authors decided the most relevant role category. There was substantial agreement between the two judges, Cohen’s κ = 0.67 (95% CI: 0.29–1.1), *p* < .001.

#### Framing of the pandemic

We used an exploratory sequential mixed-methods design (Creswell & Plano Clark, 2018) to first identify distinct ways talk show guests approached and discussed the pandemic (qualitative analysis), and then examine how often specific positions were expressed by older versus younger guests (quantitative analysis). Our analysis was based on frame theory (Goffman, 1974). According to the theory, the media “frames” topics by making certain information more salient and putting information in specific contexts (Entman, 1993). Frequently used frames have a bigger impact on political crisis management (Reuben, 2009). At the time of our study, no study had investigated the framing of the COVID-19 pandemic in German media. We therefore used qualitative thematic analysis (Braun & Clarke, 2006) to conduct an extensive, exploratory prestudy to identify distinct ways talk show guests framed the pandemic (detailed information in Supplementary Material). Some younger guests were featured only with other younger guests, while others were featured together with older guests. In contrast, all older guests were featured together with younger guests. Because being in an intergenerational context may affect how guests frame the pandemic, our analysis was based on the subsample of COVID-related episodes featuring at least one older and one younger guest (*n* = 37) as opposed to the whole sample of episodes. Using transcripts of the episodes, the first author structured the guests’ statements along the four characteristics of frames (Entman, 1993, p. 52): (1) problem definition, (2) causal interpretation, (3) moral evaluation, as well as (4) treatment recommendation, and noted whether the frame was expressed by an older or younger guest. Inductively, she generated initial codes for each individual frame and identified patterns of meaning between them to develop frame categories. She repeatedly refined and revised the themes with the senior author until the following nine categories were established: (1) *natural law of the virus*, which centers on the biological characteristics of the virus leading to high transmission if corresponding measures are not implemented; (2) *disproportionate state intervention*, which criticized disproportionate restrictions of civil liberties; (3) *population unrest*, which focused on problematic behavior within the population, and the need for the population to be “on board” to tackle the COVID-19 pandemic; (4) *suffering economy*, which highlighted the priority of the economy for society and the need for more state support; (5) *pandemic as pressure cooker*, which stressed that the COVID-19 pandemic has exacerbated poor conditions asking for immediate state support; (6) *government failure to protect*, which criticized political measures and demanded correction of wrong strategies; (7) *unfair prioritization* of social and cultural areas, which are considered forgotten over the economy, needing adequate support; (8) *limitations of government*, which argued that politicians’ hands are tied in combating the COVID-19 pandemic; and lastly (9) *the situation of older people in the pandemic*, which explicitly addressed the problematic situation of older adults (e.g., high mortality, restrictions in nursing homes). An additional independent judge coded each frame as belonging to one of the nine frame categories based on a codebook developed by the first author. There was substantial agreement between the two raters, κ = 0.770 (95% CI: 0.828–0.846), *p* < .001.

### Data Analysis

Using chi-square tests of independence, we compared the proportion of older guests’ appearance on COVID-related and other episodes. We used the descriptive statistics to examine how often older guests on COVID-related episodes were young-old versus old-old, male versus female, with versus without migration experience, with versus without a functional aid, and experts as opposed to laypeople. We also used chi-square tests of independence to compare the distribution of older and younger guests across different domains of expertise.

Using the subsample of COVID-related episodes featuring at least one older and one younger guest (*n*_frames_ = 37) and chi-square (or Fisher’s exact tests when cell sizes were below 5) with Bonferroni corrections, we compared how often older versus younger guests applied each of the nine frame categories. Each individual frame was counted only once per appearance. As some guests (*k* = 26) were invited several times, which might have an impact on frame building, we used a paired-sample *t* test to check whether there was a systematic difference in the number of invitations between younger and older guests.

Cramer’s *V* was calculated as effect size for the two-dimensional chi-square tests (0.10 = small, 0.30 = moderate, 0.50 = large effect) and φ as effect size for the one-dimensional chi-square tests (0.10–0.30 = small, 0.30–0.50 = moderate, from 0.50 = large effect). We used SPSS (version 25.0, IBM Corp., Armonk, NY) to perform all statistical analyses.

## Results

In 2020, Germany’s four most popular talk shows broadcast a total of *N* = 136 episodes, featuring a total of *K* = 754 appearances. The majority of episodes were related to COVID-19 (*n*_C19_ = 88, 64.71%) and featured *k*_C19_ = 490 guests; the other episodes (*n*_other_ = 48, 36.29%) featured *k*_other_ = 264 guests. The subsample of episodes (*n*_frames_ = 37 episodes) with all older guests in our sample featured *k*_frames_ = 183 guests.

### Underrepresentation of Older People, Particularly on COVID-Related Episodes

Older guests comprised 12.20% of all political talk show guests in 2020 (see characteristics of guests in Table 1), which is an underrepresentation compared to the German population (20.90%). Older guests appeared even *less* frequently on COVID-related than on other episodes: just 9.59% (*k*_C19_ = 47) of the guests on COVID-related episodes were 65+, compared with 17.05% (*k*_other_ = 45) of the guests featured on other episodes, χ ^2^(1, *N* = 754) = 8.89, *p* = .003, with a small effect Cramer’s *V* = 0.109. The vast majority of older guests on COVID-related episodes were male, young-old, German-born guests without migration experience, and without any visible functional aids (see Table 1).

**Table 1.** Characteristics of German Political Talk Show Guests in 2020

Characteristic	German population (81.8 M)	Episodes		
		All (*N* = 136 episodes; *K* = 754 guests)	COVID-related (*n*_C19_ = 88; *k*_C19_ = 490)	Other (*n*_other_ = 48; *k*_other_ = 264)
	% (population)	% (*N*)	% (*n*)	% (*n*)
Women	50.61% (41.4 M)	38.73% (292)	38.78% (190)	38.64% (102)
Migration experience	16.57% (13.7 M)	7.82% (59)	4.90% (24)	13.21% (35)
Functional aid	9.50% (7.9 M)	0.93% (7)	0.41% (2)	1.89% (5)
<65 years	79.10% (64.7 M)	87.80% (662)	90.41% (443)	82.95% (219)
Women	49.57% (31.6 M)	42.75% (283)	41.08% (182)	46.12% (101)
Migration experience	18.52% (11.8 M)	7.70% (51)	4.97% (22)	13.24% (29)
Functional aid	5.26% (3.4 M)	0.27% (2)	0.23% (1)	0.46% (1)
Expert	—	96.37% (638)	95.03% (421)	99.09% (217)
65+ years	20.90% (17.1 M)	12.20% (92)^a^	9.59% (47)	17.05% (45)
Young-old	68.51% (12.3 M)	93.48% (86)^b^	93.62% (44)	93.33% (42)
Old-old	31.49% (4.8 M)	6.52% (6)	6.38% (3)	6.67% (3)
Women	52.49% (9.5 M)	9.78% (9)	17.02% (8)^c^	2.22% (1)
Migration experience	10.50% (1.9 M)	8.70% (8)	4.26% (2)	13.33% (6)
Functional aid	26.32% (4.5 M)	5.43% (5)	2.13% (1)	8.89% (4)
Expert	—	95.65% (88)	91.49% (43)	100% (45)

*Notes*: Statistics on the German population from the 2019 Microcensus (Federal Statistical Office of Germany, 2020, 2021a). Functional aid includes hearing aid, walking stick, armband, wheelchair, or walker. COVID-19 = coronavirus disease 2019.

^a^There was a statistically significant difference between younger and older guests in COVID-related episodes, χ ^2^(1, *N* = 490) = 320.033, *p* ≤ .001, with a large effect φ = 0.653.

^b^Young-old guests appeared statistically significant more often in COVID-related episodes than old-old guests, χ ^2^(1, *N* = 47) = 35.766, *p* ≤ .001, with a large effect φ = 0.760.

^c^Chi-square test between older male and female guests was significant, χ ^2^(1, *N* = 47) = 20.45, *p* ≤ .001, with a medium effect, φ = 0.435.

### No Differences Between Older and Younger Guests in Professional Background

Nearly all older and younger talk show guests were experts (see Table 2). There was no differences with regard to the distribution of older and younger expert guests across politics, journalism, science, or other fields, χ ^2^(3, *k* = 464) = 1.11, *p* = .78 and χ ^2^(3, *k* = 258) = 5.84, *p* = .12. Notably, no guest represented an organization for older people. Further, there were only a few guests from the care sector including civil society, the state/bureaucracy, and health sector (*k* = 8).

**Table 2.** Professional Background of Guests on COVID-Related and Other Episodes, Overall and by Guests’ Age

Professional background	COVID episodes (*n*_C19_ = 88)			Other episodes (*n*_other_ = 48)		
	All (*k*_C19_ = 490)	Younger (*k*_<65_ = 443)	Older (*k*_65+_ = 47)	All (*k*_other_ = 264)	Younger (*k*_<65_ = 219)	Older (*k*_65+_ = 45)
	% (*k*_C19_)	% (*k*_<65_)	% (*k*_65+_)	% (*k*_other_)	% (*k*_<65_)	% (*k*_65+_)
Experts	94.72 (464)	95.03 (421)	91.49 (43)	99.24 (262)	99.09 (217)	100 (45)
Politics	33.62 (158)	32.51 (144)	32.56 (14)	46.21 (122)	43.84 (96)	57.78 (26)
Journalism	19.57 (92)	19.19 (85)	14.89 (7)	25.76 (68)	28.31 (62)	13.33 (6)
Science	17.45 (82)	16.70 (74)	17.02 (8)	4.54 (12)	5.02 (11)	2.22 (1)
Other	26.94 (132)	26.64 (118)	29.79 (14)	22.73 (60)	21.92 (48)	26.67 (0)

*Note*: COVID-19 = coronavirus disease 2019.

### Younger and Older Guests Framed the COVID-19 Pandemic Similarly—Yet Older Guests More Likely to Discuss Importance of Civil Liberties

A total of *F* = 284 individual frames were identified in the subsample of COVID-related episodes featuring at least one older and one younger guest (*n*_frames_ = 37 episodes, *k*_<65_ = 136 and *k*_65+_ = 47 guests). Most of the frames were expressed by younger guests (*f*_<65_ = 219 by younger guests; *f*_65+_ = 65 by older guests). Though the average number of frames per guest did not depend on age, younger guests: *M* = 1.30; *SD* = 0.82; older guests: *M* = 1.42; *SD* = 0.94; *t*(136) = 0.17, *p* = .76.

The most frequently occurring frame categories were *population unrest* (*f* = 54), followed by *government failure to protect public* (*f =* 36) and *the situation of older people* (*f* = 36). Thus, most often guests addressed the behavior of the population as the main problem of the COVID-19 pandemic, such as Malu Dreyer, 59 years old, chief minister of Rhineland-Palatia, on *Anne Will*, September 20, 2020:

People might lose their trust in political decisions and the situation might spiral out of control due to the wrong framework given by politicians. If the population will not stick to the measures, then the incidence rates would be too high for public health departments to follow-up cases causing fatal consequences for people with morbidities and old people. Thus, politics need to integrate the population into political decision making through better communication and calls to the population. (English translation)

There was a significant difference in the distribution of frames (see Table 3) expressed by younger and older guests across the eight frame categories, *χ*^2^(7, *F* = 284) = 19.5, *p* = .007, Cramer’s *V* = 0.281. Namely, the *disproportionate state intervention* criticizing the government restrictions on civil liberties was expressed more often by older than by younger guests, 16.93% versus 5.48% of expressed frames, *χ*^2^(1, *F* = 23) = 8.82, *p* = .003, Cramer’s *V* = 0.176. Older guests’ use of the *disproportionate state intervention* is illustrated by the following synopsis of the statements made by Hans-Ullrich Jörges, 69 years old, journalist, on *Maischberger*, May 27, 2020:

**Table 3.** Discussion of Nine Themes on COVID-Related Episodes Featuring at Least One Older and One Younger Guest, Overall and by Age of Guest (*n*_frames_ = 37 Episodes, *F* = 284 Frames)

Theme	Frames from		
	All guests *F* = 284	Younger guests *f*_<65_ = 219	Older guests *f*_65+_ = 65
	*F*	% (*f*_<65_)	% (*f*_65+_)
Population unrest	55	21.00% (46)	13.85% (9)
Government failure to protect	36	11.42% (25)	16.92% (11)
Natural law of virus	29	10.96% (24)	7.69% (5)
Pandemic as pressure cooker	28	9.13% (20)	12.31% (8)
Unfair prioritization	27	9.13% (20)	10.77% (7)
Limitations of government	25	5.02% (25)^a^	0.00% (0)^a^
Suffering economy	25	9.13% (20)	7.69% (5)
Disproportionate state intervention	23	5.48% (12)^a^	16.92% (11)^a^
Situation of older people^b^	36	12.33% (27)	13.85% (9)

*Notes*: Absolute numbers of frames in parentheses. COVID-19 = coronavirus disease 2019.

^a^Significant difference in the proportion of frames from younger and older guests based on a chi-square test of independence. Due to multiple comparisons, the difference was considered statistically significant at *p* < .006.

^b^Double coding.

Restricted civil liberties and regional differences in measures are problematic. Politics has interfered too much with fundamental rights, which is disproportionate at the current low incidence rates. There have been many negative consequences of the interventions for society. (English translation)

Furthermore, the *limitations of government* frame, excusing political decisions, was addressed more often by younger than by older guests, 5.02% versus 0.00% of expressed frames, Fisher’s exact test *p* = .004, Cramer’s *V* = 0.169. There were no other significant differences between younger and older guests. Younger guests’ use of the *limitations of government* frame is illustrated by the statement of Jens Spahn, 40 years old, Federal Minister of Health, on *Maischberger*, December 9, 2020:

It is problematic that older people are not protected in nursing homes from the virus and are locked up. The virus cannot be stopped. Politicians are doing their best, but the whole world is asking for the same equipment. There is nothing we can do now. (English translation; double-coded as the *situation of older people* frame)

Notably, older guests were no more likely to address the *situation of older people* during the COVID-19 pandemic than younger guests (about 13% of the frames adopted by older and younger guests). Remarkably, the *situation of older people* frame primarily referred to the oldest old. One of very few exceptions was the statement of Peter Tschentscher, 54 years old, mayor of the city of Hamburg on *hart aber fair* on November 9, 2020:

Older adults and risk groups are not protected sufficiently as the government has neglected shielding the older population. The majority of older adults in the city of Hamburg do not live in nursing homes, thus are not taken into account in political strategies. Measures need to take into account more than just nursing homes. (English translation)

## Discussion

Our study adds to the scant empirical evidence regarding manifestations of social participation as well as media occurrences of older adults during the COVID-19 pandemic. Our results indicate a lack of social inclusion of older adults in the media discourse on the COVID-19 pandemic. Compared to the German population (20.90%), older adults were dramatically underrepresented as guests (12.20%), and even more so on COVID-related episodes (9.59%). This underrepresentation of older adults in the media can be interpreted as a type of structural ageism (Loos & Ivan, 2018) and the increasing risk of social exclusion in old age (Walsh et al., 2021). Thus, our results reflect opinion papers (see Silva et al., 2021) on (re)emergences of societal ageism during the COVID-19 pandemic and might even be interpreted within the findings on increasing ageism over the last 200 years (Ng et al., 2021).

Further, we found significant references of intersectionality ignoring age-related challenges and the heterogeneity within the older population (Walsh et al., 2021; Ylänne, 2015). Thus, the perspectives of older people’s diverse needs and competencies were not empowered when the groups most affected by the COVID-19 pandemic (e.g., older people with low economic status, older residents in long-term care homes) did not participate in the media discourse on COVID-19. Remarkably, nearly all older guests were young-old, male experts with no visible functional impairments or migration experience, which reflects previous studies on the “healthy and active” performance of older adults in the media (e.g., Kessler et al., 2010; Ylänne, 2015). Therefore, our results may support the existence of “compulsory youthfulness” (Gibbons, 2016), whereby only older people who exemplify the notion of “successful aging” are represented in German media.

Furthermore, talk show producers—as gatekeepers to a key arena of public debate in Germany—failed to include older persons or people from organizations advocating for older adults even when the older population, particularly in nursing homes, faced multifaceted consequences in the COVID-19 pandemic. As a result, German political talk shows have not contributed to a balanced and diversified opinion making. While it has been shown that German talk show producers changed the proportion of professional fields they invited guests from due to the pandemic (with more scientists being invited; Fröhlich & Hillje, 2020), they neglected to increase the proportion of older guests as an affected group of the COVID-19 pandemic. Thus, only a few and further a small selected group of older people was included to participate in the public debate on COVID-19 pandemic.

The underrepresentation of the diverse population of older adults needs to be interpreted in concert with our finding on older guests’ self-presentations in political talk shows, as assessed by their public positions toward the COVID-19 pandemic. Overall, we found no unique position of older adults, yet older guests tended to approach the COVID-19 pandemic similarly as younger guests.

There was only one exception from the overall age-congruent pattern of positions identified in our study. In their criticism of *disproportionate state intervention*, this group of older guests emphasized individual responsibility over social interdependencies and the common good, which might be considered a strong expression of values of singularity in late modernity (Reckwitz, 2020). Further, this finding might again indicate the guests’ own rather privileged background, neglecting the reality of those in socially deprived environments (Wachtler et al., 2020). Alternatively, this might be interpreted as generational effect. Older guests might have identified themselves as social corrective within the legacy of their generation of criticizing a too omnipotent state having witnessed the country’s history in developing a democratic legislation. The German student movement in the 1960s had fostered a mindset observing democratic institutions critically (Negt, 2008). In turn, younger guests’ greater attention to the *limitations of government*—providing easy excuses for politicians’ decisions—may reflect empirical findings of lower levels of ambivalence of younger compared to older people in the COVID-19 pandemic (Falvo et al., 2021). However, these findings may also point to the fact that very few politicians aged 65+ years within the government appeared in this sample, and that guests stick to the roles they were invited for (Roth, 2015). This would again reflect other studies’ findings on the unbalanced choice of guests in talk shows (Fröhlich & Hillje, 2020; Kessler & Lachenmeier, 2017).

Lastly, older guests did not address the *situation of older people* in the COVID-19 pandemic more often than younger guests. This seemingly contradictory finding might indicate self-distancing from the frailty and loss of agency associated with the fourth age (Higgs & Gilleard, 2014) as the situation of older people in the pandemic majorly focused on the oldest old, particularly the subgroup of older people living in nursing homes. This self-directed ageism is in line with findings according to which older adults are ageist toward people older or more disabled than themselves (Dobbs et al., 2008). Alternatively, this highly selective group of older guests may have been less affected by the challenges posed by the COVID-19 pandemic due to their privileged position.

In order to include the diverse needs and perspectives of older adults in public debates a diverse representation of older adults needs to be guaranteed above all. Thus, society missed a chance to profit from these perspectives in tackling the COVID-19 pandemic.

### Limitations and Suggestions for Future Research

Our analyses were based exclusively on data from 2020. We are therefore unable to draw conclusions about how the COVID-19 pandemic may have *changed* the participation and representation of older adults on political talk shows compared to prepandemic times. In light of increasing political recognition of the need to include older people in societal decision making and the disproportionate focus on old age as a risk factor during the pandemic, we examined the participation of older guests on political talk shows. Our categorization of guests as “older” or “younger” based on their chronological age may inadvertently reinforce chronological age as a meaningful characteristic. Future research should compare the participation of older people in the pandemic-related debate with the participation of other risk groups (e.g., obese people) and social groups disproportionately affected by the pandemic (e.g., single parents). Moreover, our frame analysis was based only on the subsample of *n*_frames_ = 37 COVID-related episodes featuring at least one older and one younger guest and provides only a rough representation of the discourse. Finally, we caution that our results may not generalize to other countries, other media formats, or other arenas of public debate and social participation.

Quantitative and qualitative studies examining the representation of older people during the COVID-19 pandemic in other social fora (e.g., political parliaments, or social movements), and/or other social groups of older adults, other indictors of participation in social discourse (e.g., speech time), in other countries (e.g., low-income countries, countries with younger populations), and over time (e.g., before and after the start of the COVID-19 pandemic and/or across different stages of the COVID-19 pandemic) would provide additional insight about how older adults have contributed to shaping society’s response to the COVID-19 pandemic.

## Conclusion

Using the sample case of German political talk shows, our results suggest that one prominent part of German media failed to adequately represent the diverse voices of the older population in the pandemic year 2020. The appearance and self-presentation of a small group of rather privileged older guests did not counteract this trend. Thus, rather than advocating for their age peers, older guests approached the pandemic similarly to younger guests, with a tendency to more strongly emphasize the importance of individual responsibility.

## Supplementary Material

igac009_suppl_Supplementary_Material
