# Supplementary material for: (In)visible and (Un)heard? Older Adults as Guests on COVID-Related Political Talk Shows in Germany
Source: Innov Aging. 2022 Mar 2;6(2):igac009. doi: 10.1093/geroni/igac009 (PMC9044202; doi:10.1093/geroni/igac009)
Supplement: igac009_suppl_Supplementary_Material [file igac009_suppl_supplementary_material.docx]

**Qualitative Pre-Study**

The qualitative pre-study was conducted with the aim to understand how guests generally frame the pandemic in German talk shows, a social phenomenon that had not been studied and benefits therefore from an inductive qualitative approach – building theory on patterns found in the material. Framing theory is a prominent theoretical approach to how people organize and define the world they experience (Goffmann, 1974). Adopting framing theory in this study, we assume that certain perceptions on Covid-19 are selected and made more salient (Entman, 1993, p. 52) in the political talk shows, while others are downplayed. Therefore, the more often certain frames are applied in the public discourse, the more dominant they are in shaping the political crisis management (Reuben, 2009), such as how to best tackle the multiple consequences of the COVID-19 pandemic.

**Data collection**

We selected the pre-study study’s subsample by choosing the COVID-19 related episodes in which all older guests were invited (*n_frames_* = 37). The videos were retrieved either from the channels’ online archives or from youtube.com.

**Description of data analysis**

The first author transcribed all utterances of the guests on the talk shows watching the sample’s episodes. She conducted a thematic analysis (Clarke & Braun, 2006) to identify the patterns of meaning in the guests’ statements, which we refer to as frames. She first structured the guests’ utterances related to the COVID-19 pandemic along the four characteristics of frames (Entman, 1993, p. 52): (1) Problem definition, (2) causal interpretation, thus identifying what or who is responsible for the problem, (3) moral evaluation of the causal agents and their effects, and/or (4) treatment recommendation. Then, she familiarized herself with the data by reviewing the material and taking notes. Subsequently, she generated initial codes for each individual frame using QDA software Maxqda Analytics Pro (version 2020, VERBI) of all individual frames found (*n* = 248). Analyzing the relations and patterns of meaning of initial codes like *freedom*, *social sphere*, *economy*, *measures*, she developed detailed initial themes such as *social consequences of pandemic*, *delayed political strategy*, or *disproportionate measures*. In a next step, she refined themes. Thus, while for example the *natural law of the virus* was not essentially negated in any of the other frames, no other frame considered the natural law of the virus itself as the problem and recommended to adapt to it to tackle the pandemic. To illustrate the procedure: initial categories that appeared similarly in the first round of coding, which she grouped first as initial themes, e.g., as *social consequences of the political measures*, were identified when refining the categories as three distinct themes: *pandemic as pressure cooker*, *unfair prioritization*, and *population unrest*. Thus, while several frames contained the social consequences of the political measures, several distinctions were identified between the refined themes. While the frame *pressure cooker* perceives the pandemic has exacerbated poor conditions yet are not caused by the present political measures alone, *unfair prioritization* emphasizes the current neglect of cultural and social spheres highlighting that other (economical) spheres are preferred by the government. The *population unrest* might be considered *a social consequence of the political measures*, however, the problem focuses on the behavior of the population, not on existing poor conditions or unfair prioritization. Further, the theme does not emphasize the government failures, yet problematizes the behavior itself and its consequences for the entire society. Thus, there is no overlap with *government failure to protect*, but it constitutes a distinct frame in itself.

The first author and the senior author revised and defined the themes repeatedly critically engaging with the material until they defined and named eight themes, i.e., meta frames.

At a later stage, we were interested whether the frames changed over the course of the Covid-19 pandemic in 2020. Therefore, the first author being familiar with the frames took notes on the date of the episodes. Deductively she developed the code *first lockdown* (*n* = 61) (defined as first episode in the sample until May, 4 2020 as point in time when the lockdown was officially declared to be over), the brief reprise of measures in *summer (n = 26)* (the exact dates of August, 5 2020 until September, 20 2020 were developed inductively)*,* and *lockdown light* (*n* = 72) (from October 28 2020) when the German government announced the lockdown light until the end of 2020). Inductively, she coded *post-first lockdown* (*n* = 46) (after the first lockdown until July), and *fall* (*n* = 35) (September, 21 2020 until October, 28 2020), as well as the exact dates of *summer* by distinguishing between episodes that referred to the previous time (*post-first lockdown*) and the upcoming time (*fall*).

**Results**

All the frames generated address the COVID-19 pandemic, yet, they frame the pandemic differently, thus highlighting certain aspects, while downplaying other aspects that are more prevalent in the other frames: (1) *Natural law of the virus*, (2) *disproportionate state intervention*, (3) *population unrest*, (4) *suffering economy*, (5) *pandemic as pressure cooker*, (6) *government failure to protect*, (7) *unfair prioritization*, (8) *limitations of government*. A remaining category contained individual frames (*n* = 9) that contained highly unique positions, which could not be coded as one of the eight meta frames. Furthermore, the first author generated frames that centered around the difficulties for older people in the pandemic in the initial coding. This frame of (9) *the situation of older people* in the pandemic can be considered an umbrella frame as there is more variety in the individual frames coded. Thus, the individual frames were double-coded as well as one of the eight frames as they were more coherent in those frames. Positions of the frame the *situation of older people* in the pandemic (*n* = 36) were double-coded majorly in the following frames: *government failure to protect* (*n* = 11; 30.5%), *natural law of the virus* (*n* = 7; 19.4%); *limitations of government* (*n* = 6; 16.7%), and *pandemic as pressure cooker* (*n* = 5; 13.9%). This frame addresses the problematic situation of older people either due to high mortality rates, or social isolation in nursery homes. Majorly this is attributed to political failure. Morally, this is primarily considered a catastrophe as it should have been prevented. Utilizing protective equipment for long-term care and all nursery homes, e.g., is one of the treatment recommendation in this frame.

(1) *Natural law of the virus* was primarily used during *lockdown light* (55%) when the case numbers and mortality rates increased drastically. The frame centers on the biological characteristics of the virus leading to high transmission if corresponding measures are not taken. (2) *Disproportionate state intervention* criticized disproportionate restrictions of civil liberties and was used almost equally (around 20%) in *post-first lockdown,* in *fall* and during *lockdown light.*  The frame (3) *population unrest* focusing on problematic behavior within the population, which should be “on board” to solve the COVID-19 pandemic. It was used almost equally used over the entire time with slight peaks in *lockdown light* (25%) when the incidence rate was high and the compliance with measures was debated, and in *summer* (24%) when the parts of the population demonstrated on the measures. The (4*) suffering economy* stressed the economy’s priority for society demanding more state support, mostly debated in the beginning of the Covid-19 pandemic in Germany in the *first lockdown (*39%*)*, yet was framed to be a problem throughout the different phases in the pandemic. The frame (5) *pandemic as pressure cooker* stresses that the COVID-19 pandemic aggravated poor conditions for vulnerable groups was mostly used in the beginning of the pandemic during the *first lockdown* and *post-first lockdown* (taken together 79%) and was not thematized afterwards strongly in the talk shows. The frame (6) *government failure to protect* criticizes political measures and demands correction of wrong strategies, primarily used in the *first lockdown* (29%) and *lockdown light* (50%), when the incidence rates were high and the type of political measures were debated.

The (7) *unfair prioritization* of social and cultural areas, which are considered forgotten over the economy needing adequate support, were framed as problem primarily in the beginning of the pandemic in the *first lockdown, post-first lockdown* (56%) as well as during *lockdown light* (33%). The (8*) limitations of government,* arguing that the hands of politicians are tied in combatting the COVID-19 pandemic, appeared early on in the *first lockdown (35%)* and was used during *lockdown light (39%)* at a similar frequency*.* Lastly, *(9) the situation of older people in the pandemic* addressed the problematic situation of older adults such as high mortality rates or restrictions in nursing home. Guests used this frame mostly in *fall* (33%) and *lockdown light (36%)* discussing that the dramatic situation of the first lockdown should not repeat itself, as well as in the *first lockdown* (*28%*) when high mortality rates and social isolation was discussed for the first time, and just once in *summer*. Focusing on the time-related aspects of the frame, we found that all frames appear throughout the different phases of the year 2020 in our sample with the exception of the frame *pressure cooker* on the worsened situation for vulnerable groups that was framed as problem mostly at the beginning of the Covid-19 pandemic.

In the following table, there are examples for each frame.

| Frame | Example |
| --- | --- |
| Natural law of the virus | A huge problem is the uncontrolled transmission of the virus as the population does not adhere to the measures accordingly. Consequentially, hospitals are overburdened, high mortality rates of older people and at-risk groups. There is no alternative to a strict shutdown, otherwise the situation will be out of control (Viola Priesemann, 38 years, physicist on Anne Will, November 1, 2020). |
| Disproportionate state intervention | Political measures are disproportionate and cause concern, as the current incidence numbers are low. Government is to blame as it prioritizes protection over freedom, which creates a nation in standstill. The foundation for the restrictions are unclear and the consequences of the measures are severe. Demanding a quick easing of the measures and regionally differentiated measures (Christian Lindner, 41 years, politician in the opposition party FDP [Free Democratic Party of Germany], on Maybrit Illner, April 30, 2020). |
| Population unrest | Of great concern are insecurity, existential fear, displeasure in parts of the population. Politics has not taken up the right measures leading to aggravated imbalances. Concerns of the population must be taken seriously by politics and discussed publicly (Sahra Wagenknecht, 51 years, politician of the opposition party Die Linke [The Left] on Anne Will, May 17, 2020). |
| Suffering economy | The economy is suffering as there is no clear political concept by the government to tackle the pandemic. There has always been risks in life, so politics should intervene less, and ease the restrictions soon. There needs to be more innovation and support in the economy to survive the current crisis. As a result the rest of society will come out of the crisis as well (Friedrich Merz, 65 years, politician, Government party CDU [Christian Democratic Union of Germany], on maischberger.die woche, May 27, 2020). |
| Pandemic as pressure cooker | There has been an increase in domestic violence and women and children are exposed even more strongly to abusive behavior. The facilities are overcrowded and there is fear of transmission. This is a difficult situation for the affected people, which can be solved with financial and pedagogic support for children and women (Jutta Speidel, 66 years, actress, on maischberger.die woche, April 1, 2020). |
| Government failure to protect | Politicians applied the wrong strategy as they failed to included at-risk groups or take into account social and economic consequences. It is a constant back and forth in political measures. There needs to be a public debate on risk assessment and as consequence shielding for older people, proving masks, contact tracking, fast responses to new cases, and more testing facilities (Alexander Kekulé, 62 years, virologist, on maischberger.die woche, April 29, 2020). |
| Unfair prioritization | There is too much pressure on theaters since the government has restricted the cultural sphere unfairly. The restrictions are unfair as other sectors such as brothels are open. Concrete solutions to overcome the tight situation need to be provided by politicians so theaters can be opened again (Dieter Hallervorden, 85 years, Comedian, on hart aber fair, September, 21 2020). |
| Limitations of government | It is problematic that parents are dissatisfied with how government dealt with home schooling. However, there is no one to blame since no one had any idea how to solve it. The situation can be seen as learning experience for everyone and we need to protect teachers as they are blamed by parents (Susanne Eisenmann, 55 years, politician in the government party CDU [Christian Democratic Union of Germany] on hart aber fair, May 25, 2020). |
| Situation of older people | Older people have been harmed disproportionately by political measures and were locked away. We used to quarantine the ill, yet nowadays older people are locked away so younger people can enjoy their lives. We need to shield old and vulnerable people in a more humane way (Frank Ulrich Montgomery, 68 years, president of the World Medical Association, on Maybrit Illner, December 10, 2020). |
